# Dosage and duration effects of nitrogen additions on ectomycorrhizal sporocarp production and functioning: an example from two N-limited boreal forests

**DOI:** 10.1002/ece3.1145

**Published:** 2014-07-05

**Authors:** Niles J Hasselquist, Peter Högberg

**Affiliations:** Department of Forest Ecology and Management, Swedish University of Agricultural Sciences (SLU)Skogsmarksgränd, Umeå, SE-901 83, Sweden

**Keywords:** Forest soils, mycorrhiza, nitrogen limitation, nitrogen retention, nitrogen stable isotope, Scots pine, sporocarp production, symbiosis

## Abstract

1. Although it is well known that nitrogen (N) additions strongly affect ectomycorrhizal (EM) fungal community composition, less is known about how different N application rates and duration of N additions affect the functional role EM fungi play in the forest N cycle.
2. We measured EM sporocarp abundance and species richness as well as determined the *δ*^15^N in EM sporocarps and tree foliage in two *Pinus sylvestris* forests characterized by short- and long-term N addition histories and multiple N addition treatments. After 20 and 39 years of N additions, two of the long-term N addition treatments were terminated, thereby providing a unique opportunity to examine the temporal recovery of EM sporocarps after cessation of high N loading.
3. In general, increasing N availability significantly reduced EM sporocarp production, species richness, and the amount of N retained in EM sporocarps. However, these general responses were strongly dependent on the application rate and duration of N additions. The annual addition of 20 kg·N·ha^−1^ for the past 6 years resulted in a slight increase in the production and retention of N in EM sporocarps, whereas the addition of 100 kg·N·ha^−1^·yr^−1^ during the same period nearly eliminated EM sporocarps. In contrast, long-term additions of N at rates of *ca*. 35 or 70 kg·N·ha^−1^·yr^−1^ for the past 40 years did not eliminate tree carbon allocation to EM sporocarps, although there was a decrease in the abundance and a shift in the dominant EM sporocarp taxa. Despite no immediate recovery, EM sporocarp abundance and species richness approached those of the control 20 years after terminating N additions in the most heavily fertilized treatment, suggesting a recovery of carbon allocation to EM sporocarps after cessation of high N loading.
4. Our results provide evidence for a tight coupling between tree carbon allocation to and N retention in EM sporocarps and moreover highlight the potential use of *δ*^15^N in EM sporocarps as a relative index of EM fungal sink strength for N. However, nitrogen additions at high dosage rates or over long time periods appear to disrupt this feedback, which could have important ramifications on carbon and nitrogen dynamics in these forested ecosystems.

## Introduction

It is well understood that mycorrhizas, the symbiotic associations between fungi and plant roots, are ubiquitous features of nearly all terrestrial plant communities (Smith and Read 2008). Mycorrhizal fungi efficiently explore the soil to acquire nutrients, some of which is transferred to host plants in exchange for recently fixed carbon (C). In forested ecosystems where nitrogen (N) is a major limiting nutrient, such as boreal forests, it is thought that ectomycorrhizal (EM) fungi play a key role for host plant N acquisition and tree growth (Read et al. 2004; Smith and Read 2008; but see Näsholm et al. 2013). However, increasing use of fertilizers and anthropogenic N deposition from agricultural and industrial emissions has drastically affected the global N cycle (Vitousek et al. 1997; Galloway et al. 2008), and, as a result, many ecosystems previously characterized by N limitation have experienced an increase in N availability. Increasing N availability in natural ecosystems has major ecological implications, including, but not limited to, changing biological diversity, acidifying soils, altering the global C cycle, and changing the rate and pathways of N cycling (Tilman 1987; Aber et al. 1995; Vitousek et al. 1997; Chung et al. 2007; Galloway et al. 2008).

Trees decrease belowground C allocation in response to increasing soil N availability (Ågren and Franklin 2003; Mäkelä et al. 2008; Franklin et al. 2009), which results in reduced C supply to EM fungi in particular (Wallenda and Kottke 1998; Högberg et al. 2003; Treseder 2004; Högberg et al. 2010). Numerous field studies have shown a reduction in C allocation to EM fungi in response to elevated N deposition and N additions (Arnolds 1991; Wallander and Nylund 1992; Termorshuizen 1993; Arnebrant 1994; Wallenda and Kottke 1998; Lilleskov et al. 2001; Nilsson and Wallander 2003; Nilsson et al. 2005, 2007; Ostonen et al. 2011; Kjoller et al. 2012), with changes in aboveground sporocarp production being more sensitive to N supply than changes belowground. It is also well recognized that increases in soil N availability reduce EM species richness and cause significant changes in EM fungal community composition (Lilleskov et al. 2001, 2002a,b2002b; Avis et al. 2003; Toljander et al. 2006; Parrent and Vilgalys 2007; Cox et al. 2010; Lilleskov et al. 2011; Kjoller et al. 2012). In short, the results show an increase in *Lactarius*, *Laccaria*, and *Paxillus* species with increasing N additions (i.e., “nitrophilic” taxa sensu Lilleskov et al. 2001), whereas *Cortinarius*, *Suillus*, *Tricholoma,* and *Piloderma* species respond negatively to elevated N (i.e., “nitrophobic” taxa sensu Lilleskov et al. 2001). Despite our increasing knowledge of the impacts N additions have on EM fungal community composition, less is known about how different N application rates and duration of N additions affect the functional role EM fungi play in the forest N cycle. Furthermore, the recovery of EM sporocarps after the termination of long-term N additions remains unclear.

Because of the tight coupling between C and N in mycorrhizal symbioses, changes in tree C allocation to EM fungi can have dramatic effects on the transfer of N in EM symbioses (Alberton et al. 2007; Näsholm et al. 2013). Although the common dogma is that N is readily transferred from EM fungi to host plants in environments characterized by low N availability, several pot studies (Colpaert et al. 1996; Alberton et al. 2007; Correa et al. 2008; Alberton and Kuyper 2009) as well as a recent field study (Näsholm et al. 2013) have demonstrated that EM fungi retain large amounts of N. Using a ^15^N tracer technique, Näsholm et al. (2013) showed that greater C allocation to EM fungi in an N-limited boreal forest resulted in increased N retention in EM fungal biomass, whereas the addition of N fertilizer reduced C allocation to EM fungi and shifted the incorporation of ^15^N tracer from EM fungi to tree foliage. Thus, changes in C allocation to EM fungi in response to increased N availability could directly affect soil C dynamics (Clemmensen et al. 2013) in addition to having cascading effects on the cycling of N in forest soils (Högberg et al. 2011; Nilsson et al. 2012; Näsholm et al. 2013).

In addition to ^15^N tracer studies, the ^15^N:^14^N ratio (*δ*^15^N) of EM sporocarps and tree foliage at natural abundance levels may provide a unique opportunity to explore the in situ movement of N in EM symbioses (e.g., Hobbie and Colpaert 2003; Hobbie and Hobbie 2006; Högberg et al. 2011). In general, EM fungi tend to be enriched in ^15^N under N-limited conditions, whereas foliage of host plants is depleted in ^15^N (Gebauer and Dietrich 1993; Högberg et al. 1996, 1999; Hobbie and Colpaert 2003; Hobbie 2006; Hobbie and Hobbie 2008). Differences in ^15^N signatures between EM fungi and their host appear to be the result of fractionation of roughly 8–10 ‰ during the creation of transfer compounds (Macko et al. 1986; Hobbie and Colpaert 2003), with preferential retention of the heavier ^15^N isotope by EM fungal biomass and the transfer of the lighter ^14^N isotope to host plants (Taylor et al. 1997; Högberg et al. 1999; Kohzu et al. 2000). Previous studies, with a phytocentric perspective, have used these differences in *δ*^15^N to estimate the fraction of N in trees that enters through mycorrhizal fungi (Hobbie and Hobbie 2006, 2008; Yano et al. 2010; Mayor et al. 2012; Pena and Polle 2014). In this study, we take a more mycocentric perspective and evaluate whether changes in the *δ*^15^N signature of EM sporocarps in response to N additions may be used as a relative index of EM fungal sink strength for N, with higher ^15^N values indicating greater amounts of N retained in EM sporocarp biomass.

This study was undertaken to examine the short-term (<6 years) and long-term (>40 years) effects of N addition on EM functioning in two oligotrophic *Pinus sylvestris* L. forests in northern Sweden. Both sites are characterized by multiple N addition treatments, which allowed us to examine the response of EM sporocarp production and species richness to different N application rates and to compare short with long-term responses. Additionally, after 20 and 39 years of N additions, respectively, two of the N addition treatments in the long-term N addition experiment were terminated, thereby providing a unique opportunity to examine the recovery of EM sporocarps after the cessation of high N loading. In addition to measuring cumulative EM sporocarp biomass and species richness, we also determined the N% and *δ*^15^N in EM sporocarps and tree foliage within the different N treatments to better understand how N additions affect the functional role EM fungi play in the forest N cycle. We hypothesize that (i) N additions will have a negative effect on EM sporocarp biomass and species richness, (ii) changes in EM sporocarp production will depend on the amount and duration of N additions, and (iii) EM sporocarp *δ*^15^N values are correlated with the cumulative amount of N retained in EM sporocarp biomass and decrease with increasing N availability. We further hypothesize that after the termination of long-term N additions, the abundance, richness, and *δ*^15^N values of EM sporocarps as well as foliar *δ*^15^N will become more similar to the control than in ongoing N addition treatments, indicating a recovery of the functional role EM fungi play in the forest N cycle after the cessation of high N loading.

## Materials and Methods

### Study sites

Two boreal *Pinus sylvestris* L. forests in northern Sweden were selected for the study. The first experimental forest site was located near Norrliden (64°21′N, 19°45′E) and was planted in 1953 after prescribed burning in 1952 (Tamm et al. 1999). The soil is a glacial till with sand as the dominant fraction. The N treatments started in 1971 and consisted of three annual addition rates of NH_4_NO_3_: 35 (N1), 70 (N2T), and 110 kg·N·ha^−1^·yr^−1^ (N3T), respectively, and a control (N0) receiving ∼2 kg·N·ha^−1^ yr^−1^ by natural deposition (*n* = three 30 × 30 m plots per N treatment). The amount of N added to these plots has varied slightly over the years and is summarized in Högberg et al. (2011). In 1991, after 20 years of N additions, the highest N treatment (N3T) was terminated, and in 2009, the N2T treatment was also terminated. More details of soil properties among the treatments are given in Table 1.

**Table 1:** The cumulative amount of N added, percent N (%), *δ*^15^N (‰) and C:N ratio of the organic mor-layer, and foliar N (%) and *δ*^15^N in different N addition treatments in two *Pinus sylvestris* boreal forests in northern Sweden. At Rosinedalsheden, N addition treatments started in 2006 and consisted of two treatments: a low (20 kg·N·ha^−1^·yr^−1^) and high N additions (100 kg·N·ha^−1^·yr^−1^), and an unfertilized control. At Norrliden, N addition treatments began in 1971 and consistent of three annual addition rates of NH_4_NO_3_: 35 (N1), 70 (N2T), and 110 kg·N·ha^−1^·yr^−1^ (N3T), respectively, and a control (N0). Significant differences (*P* < 0.05) among N treatments are indicated by different letters.

Site	Nitrogen treatment		
Rosinedalsheden	Control^1^	Low	High
Cumulative N added (kg·N·ha^−1^)		120	600
Nitrogen (%) in the mor-layer	0.97 ± 0.01	1.17 ± 0.05	1.20 ± 0.02
*δ*^15^N (‰) in the mor-layer	−0.99 ± 0.34	−1.06 ± 0.34	−0.95 ± 0.40
C:N ratio in the mor-layer	44.83 ± 2.20	39.10 ± 0.54	38.38 ± 0.45
Foliar N (%)	1.13 ± 0.03	1.23 ± 0.07	2.20 ± 0.24
Foliar *δ*^15^N (‰)	−5.16 ± 0.90	−4.73 ± 0.46	−1.94 ± 0.22

Norrliden	N0^1^	N1	N2T	N3T

Cumulative N added (kg·N·ha^−1^)		1320	2460	2070
Nitrogen (%) in the mor-layer	0.96 ± 0.03^a^	1.45 ± 0.09^b^	1.58 ± 0.07^b^	1.41 ± 0.11^b^
*δ*^15^N (‰) in the mor-layer	−0.01 ± 0.25	−0.83 ± 0.17	−0.16 ± 0.13	−0.07 ± 0.17
C:N ratio in the mor-layer	45.31 ± 1.33^a^	33.54 ± 2.42^b^	28.64 ± 0.51^b^	30.61 ± 0.22^b^
Foliar N (%)	1.21 ± 0.01^a^	1.42 ± 0.03^b^	1.54 ± 0.02^c^	1.38 ± 0.03^b^
Foliar *δ*^15^N (‰)	−3.13 ± 0.13^a^	−0.85 ± 0.25^b^	0.44 ± 0.14^c^	−2.06 ± 0.40^a^

1 Control treatments at both sites received only ambient deposition of N (∼2 kg·N·ha^−1^·yr^−1^).

The other site was located in a homogenous 70-years-old *P. sylvestris* stand 50 km northwest of Umeå at the Rosinedalsheden experimental forest (64°10′N, 19°45′E). The soil is fine sand, and the profile formed is that of a weakly developed podsol. At this site, three 15 ha plots were selected based on similar stand structure for a large-scale eddy covariance study examining the effects of N additions on net ecosystem CO_2_ exchange with the atmosphere. Although these N treatments were unreplicated due to logistical and financial constraints, they provide insights into ecosystems processes at a spatial scale which would be impossible to achieve with smaller scale, more easily replicated manipulations (Carpenter 1996; Sullivan 1997; Osmond et al. 2004). Starting in 2006, each plot has been subjected to different N treatments: a low and high N addition plots that receive annual additions of NH_4_NO_3_ of 20 and 100 kg·N·ha^−1^·yr^−1^, respectively, and an unfertilized control plot that receives ∼2 kg·N·ha^−1^·yr^−1^ by natural deposition. The amount of N added to the high N plot is typical of many scientific N addition experiments (see Hasselquist et al. 2012) and is used here to indicate the sensitivity of the system to N additions. In contrast, the dose in the low N plot was designed to mimic potential future N deposition rates in southern Scandinavia according to model projections (Lamarque et al. 2005). More details of soil properties among the treatments are given in Table 1.

### Ectomycorrhizal sporocarp and tree foliage measurements

Within each of the N treatment plots (30 × 30 m) at Norrliden, a 10 × 10 m plot was installed for the collection of EM sporocarps. At Rosinedalsheden, we installed three 10 × 10 m plots within each of the three 15 ha N treatment plots. Thus, at both forest sites, EM sporocarps were collected from three 100 m^2^ plots per N treatment. Plots were sampled weekly throughout the summer, but most samples were collected between August and September, as this period corresponds with most EM sporocarp emergence. All fully developed EM sporocarps were collected and brought back to the laboratory where they were identified. At the end of the growing season, current-year needles were collected from the top whorls of 10 trees within each 100 m^2^ plot at both sites for nutrient and isotopic analyses. Additionally, five soil samples were collected in each plot using a 2.5-cm-diameter soil corer for nutrient and isotopic analyses of the organic mor-layer.

### Preparation and sample analyses

Samples were dried (70°C, 48 h), weighed, and then ground in a ball mill (MM200, Retsch GmbH, Haan, Germany). Thus, dry weight and number of EM fungal sporocarps collected per species were recorded each week. If multiple sporocarps of the same EM species were collected in a plot on a given sampling date, then they were pooled prior to isotopic analyses. Dried samples of sporocarp caps, soil mor-layer, and foliage were analyzed for ^15^N, %N, and %C using an elemental analyzer (Flash EA 2000) coupled to an isotope ratio mass spectrometer (Delta V Advantage, Thermo Scientific, Bremen, Germany). Results are expressed in the standard notation (*δ*^15^N) in parts per thousands (‰) relative to atmospheric N_2_. The standard deviation based on analysis of replicated samples was <0.1‰ for *δ*^15^N.

### Statistical analyses

The following community indices were calculated for each N treatment: 1) EM taxon richness (S), that is, the number of species identified, and 2) cumulative sporocarp abundance and biomass. Because many EM species were unequally represented among the N treatments and it is well known that EM species differ greatly in *δ*^15^N (Taylor et al. 2003; Hobbie and Agerer 2010), this created a potential bias when calculating mean values for the different N treatments. We therefore calculated weighted means (i.e., community means) that took this into account. Community *δ*^15^N mean values were determined separately for each plot by multiplying the relative abundance of each EM genera by the *δ*^15^N values for that particular EM genus and summing these values for all EM genera found within a plot. Similarly, the cumulative amount of N retained in EM sporocarps was calculated for each plot by multiplying the nitrogen concentration for each EM genera by the cumulative biomass for that particular genus and summing these amounts for all EM genera found within a plot.

At Norrliden, community indices and EM community *δ*^15^N values were subjected to a one-way ANOVA followed by post hoc Tukey's tests to determine significant (*P* < 0.05) differences among N treatments. For data that did not meet the assumption of homogeneity of variance, we used post hoc Games–Howell tests to determine significance. At each site, a two-way ANOVA followed by post hoc Tukey's tests was used to detect significant (*P* < 0.05) differences in *δ*^15^N of EM sporocarps at the generic taxonomic level, N addition treatments, and the interaction between EM genera and N addition treatments. Because of the limited number of *Boletus*, *Suillus,* and *Xerocomus* taxa collected, we grouped these species into Boletales, which represents a higher taxonomic level, prior to statistical analysis. For EM genera collected at Norrliden, we also performed a one-way ANOVA followed by post hoc Tukey's tests to determine significant differences among N addition treatments. Given that the N treatments were unreplicated at Rosinedalsheden, we did not perform statistical analyses to determine differences among treatments. Instead, we present treatment means (±SE) derived from the three plots to provide an estimate of within-plot variability as a guide to how robust the measured mean differences were among treatments.

Because foliar N concentrations provide a useful index to asses soil N availability (Aerts and Chapin 2000; Hobbie and Gough 2002; Cox et al. 2010), we used foliar %N data to examine how changes in N availability affect cumulative EM sporocarp biomass, species richness, and community *δ*^15^N values. Least square regression was also used to look for significant relationships among cumulative EM sporocarp biomass, EM community *δ*^15^N values, and the cumulative amount of N retained in EM sporocarps. All statistical analyses were performed using SPSS statistical software (SPSS 21.0 for windows, SPSS Inc., Chicago, IL).

## Results

### EM sporocarp production and richness among N treatments

**Figure 1:**
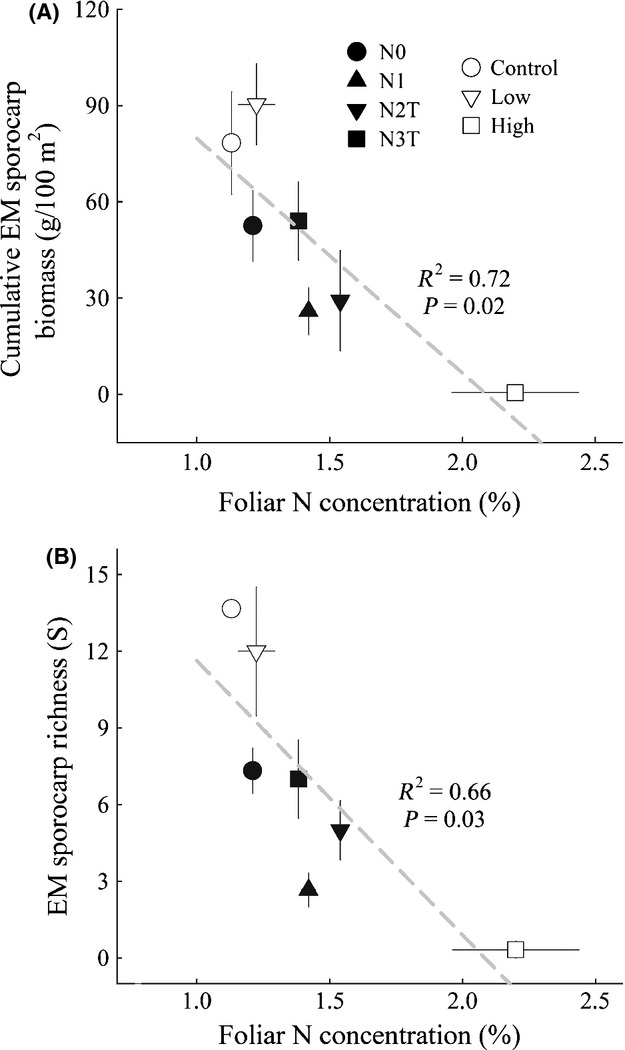
Correlation between treatment means of foliar N and cumulative EM sporocarp biomass (A) and EM sporocarp richness (B) at Norrliden (filled symbols) and Rosinedalsheden (open symbols). Data points represent the mean ±SE (*n* = 3).

**Figure 2:**
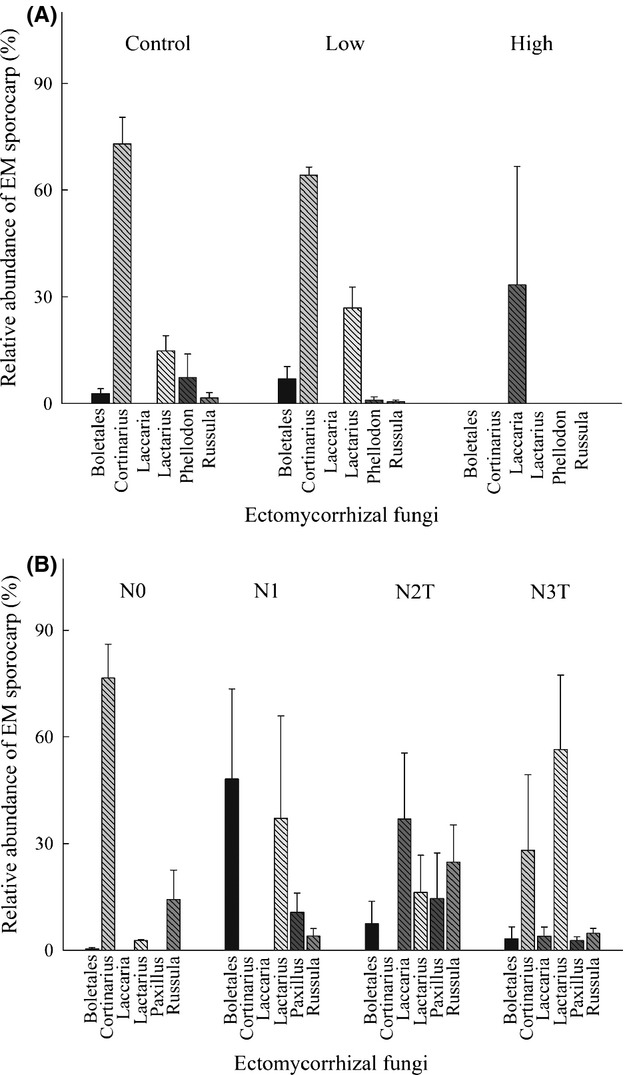
Mean (±SE) relative abundance of EM sporocarp taxa at Rosinedalsheden (A) and Norrliden (B). Relative abundance was calculated for each plot as the number of each EM sporocarp taxa from the total number of EM sporocarps collected (*n* = 3 per N treatment).

A total of 1657 EM sporocarps representing 11 genera and 33 species were collected in this study. Across both sites, cumulative EM sporocarp biomass and species richness significantly decreased with increasing N availability, as measured by foliar N concentrations (Fig. 1). At Rosinedalsheden, sporocarp production and species richness were similar between the control and low N treatment (Table 2), with *Cortinarius* species being the dominant genus in both treatments (Fig. 2A). In contrast, only 12 EM sporocarps were collected in the high N treatment, and these were found in only one plot. Moreover, all 12 sporocarps in the high N treatment were *Laccaria bicolor* (Maire) Orton, whereas *L. bicolor* was not found in the other two treatments (Fig. 2A). A complete list of EM sporocarps found among the N treatments at Rosinedalsheden is shown in Supporting Information Table S1.

**Table 2:** Comparison of EM sporocarp taxon richness (S), cumulative EM sporocarp abundance, biomass and amount of N retained in EM sporocarps, EM community C:N ratio and *δ*^15^N, and the ^15^N enrichment between EM sporocarps and tree foliage (Δ) among the different N treatments at Norrliden and Rosinedalsheden. Values shown are average ± SE (*n* = 3). Significant differences (*P* < 0.05) among N treatments are indicated by different letters.

Site	Nitrogen treatment		
Rosinedalsheden	Control	Low	High
Taxon richness (S)	13.67 ± 0.33	12.00 ± 2.52	0.33 ± 0.33
Cumulative abundance (ind per 100 m^2^)	162.00 ± 38.97	193.33 ± 50.83	4.00 ± 4.00
Cumulative biomass (g per 100 m^2^)	78.34 ± 16.06	90.40 ± 12.6	0.57 ± 0.57
Cumulative EM sporocarp N (g N per 100 m^2^)	2.85 ± 0.59	3.38 ± 0.48	0.04 ± 0.04
EM community C:N ratio	12.55 ± 0.12	12.45 ± 0.10	7.22
EM community *δ*^15^N (‰)	5.60 ± 0.19	5.30 ± 0.29	0.46
Δ (^15^N_sporocarps_ − ^15^N_foliage_)	10.78 ± 0.27	10.03 ± 0.74	2.49

Norrliden	N0	N1	N2T	N3T

Taxon richness (S)	7.33 ± 0.88^a^	2.67 ± 0.67^b^	5.00 ± 1.16^a,b^	7.00 ± 1.53^a,b^
Cumulative abundance (ind per 100 m^2^)	58.67 ± 17.15	13.33 ± 3.18	33.33 ± 14.75	87.67 ± 39.31
Cumulative biomass (g per 100 m^2^)	52.56 ± 11.04	25.93 ± 7.35	29.19 ± 15.68	54.07 ± 12.16
Cumulative EM sporocarp N (g N per 100 m^2^)	1.90 ± 0.40	1.40 ± 0.47	1.37 ± 0.72	2.20 ± 0.46
EM community C:N ratio	12.91 ± 0.30^a^	9.15 ± 0.97^b^	10.09 ± 0.12^b^	11.39 ± 0.17^a,b^
EM community *δ*^15^N (‰)	7.30 ± 0.17^a^	3.98 ± 0.70^b^	3.23 ± 1.02^b^	4.78 ± 1.23^a,b^
Δ (^15^N_sporocarps_ − ^15^N_foliage_)	10.43 ± 0.28^a^	4.83 ± 0.60^b^	2.79 ± 1.13^c^	6.84 ± 1.63^a^

At Norrliden, the ongoing addition of 35 kg·N·ha^−1^·yr^−1^ in the N1 treatment severely reduced EM sporocarp abundance and richness relative to the control, N0 treatment (Table 2). Three years after the termination of N additions in the N2T treatment, EM sporocarp abundance and richness was still reduced compared with the control. Richness and cumulative EM sporocarp biomass in the N3T treatment was more similar to the control compared with the N1 and N2T treatments. In the control, roughly 80% of all EM sporocarps collected were *Cortinarius* species (Fig. 2B). In contrast, the two most abundant EM sporocarp taxa in the N1 treatment were Boletales and *Lactarius*. It is worth pointing out that Boletales in the N1 treatment consisted entirely of *Xerocomus subtomentous* (L.) Quel., whereas Boletales in the other N treatments were characterized by *Suillus variegatus* (Swartz ex Fr.) O. Kuntze. The most abundant EM sporocarp taxon in the N2T treatment was *Laccaria*, whereas the recovery of EM species richness in the N3T treatment was largely the result of the re-occurrence of *Cortinarius* species. A complete list of EM sporocarps found among the N treatments at Norrliden is shown in Supporting Information Table S2.

### EM community and foliar ^15^N among N treatments

**Figure 3:**
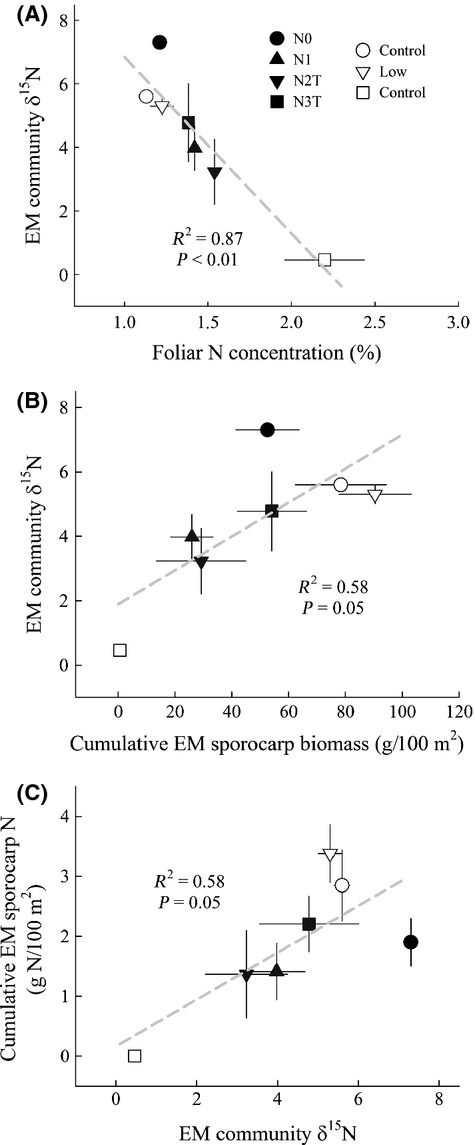
Correlation between treatment means of foliar N and EM community *δ*^15^N (A), cumulative EM sporocarp biomass and EM community *δ*^15^N (B), and EM community *δ*^15^N and the cumulative amount of N retained in EM sporocarps (C) at Norrliden (filled symbols) and Rosinedalsheden (open symbols). Data points represent the mean ±SE (*n* = 3).

Across both sites, EM community *δ*^15^N values were negatively correlated with increasing N availability (Fig. 3A), yet positively correlated with increasing cumulative EM sporocarp biomass (Fig. 3B). Higher EM community *δ*^15^N values were in turn significantly correlated with greater cumulative N retained in EM sporocarps (Fig. 3C).

After 6 years of N additions at Rosinedalsheden, *δ*^15^N of current-year needles differed little between the control and low N treatment, yet were ∼3‰ higher in the high N treatment (Table 1). EM community *δ*^15^N values were similar between the control and low N treatment, whereas for the one plot in the high N treatment in which we were able to calculate an EM community *δ*^15^N value, it was ∼5‰ lower compared with the control and low N treatment (Table 2). Taken together, differences in *δ*^15^N between EM sporocarps and tree foliage (Δ) differed little between the control and low N treatment, whereas Δ was much smaller in the high N treatment.

At Norrliden, *δ*^15^N of current-year needles was significantly lower in the control and N3T treatment compared with the N1 treatment, which was significantly lower relative to the N2T treatment (Table 1). EM community *δ*^15^N values were lowest in N1 and N2T treatments, intermediate in the N3T treatment, and highest in the control (Table 2). Taken together, Δ was greatest in the control and N3T treatment, followed by the N1 treatment, and lowest in the N2T treatment.

## ^15^N of EM sporocarps

**Figure 4:**
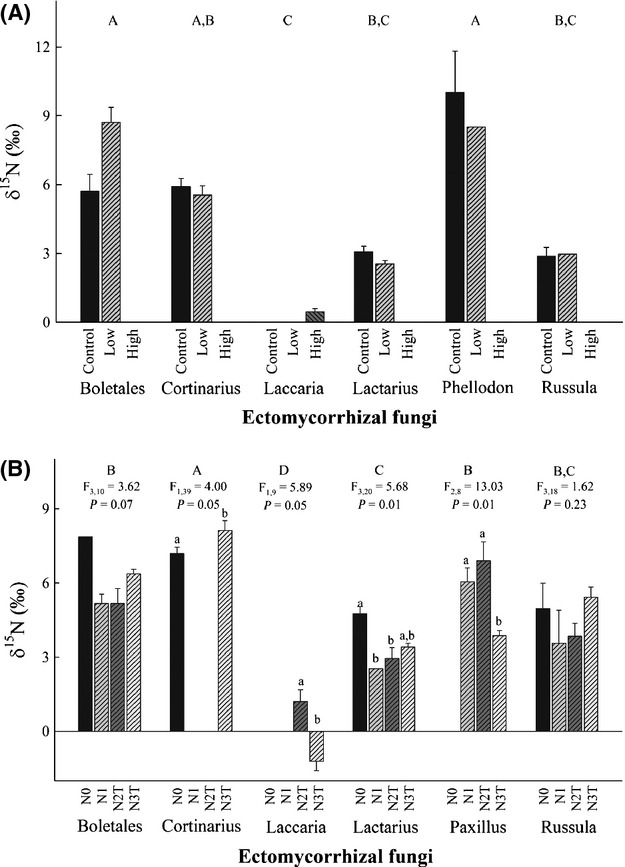
Mean (±SE) *δ*^15^N values of EM genera found within the different N addition treatments at Rosinedalsheden (A) and Norrliden (B). Different capital letters at the top of each graph represent significant (*P* < 0.05) differences among EM taxa. For each EM taxa at Norrliden, *F* and *P* values (with df) are from a one-way ANOVA testing for the effect of N addition with significant differences indicated by different lower-case letters.

At both sites, *δ*^15^N of EM sporocarps differed significantly among genera (Fig. 4), whereas differences among N addition treatments were only marginally significant at Norrliden (*P* = 0.08). For EM taxa collected throughout all the N treatments at Norrliden (i.e., Boletales, *Lactarius,* and *Russula*), sporocarp *δ*^15^N values were lowest in the N1 and N2T treatments, whereas sporocarp *δ*^15^N in the N3T treatment approaching those in the control (Fig. 4B). In contrast, *δ*^15^N values of *Laccaria* and *Paxillus* were lowest in the N3T treatment relative to the other N addition treatments.

## Discussion

Results from this study clearly show a reduction in the amount of C allocated to and N retained in EM sporocarps with increasing N availability in two oligotrophic boreal pine forests. However, this response was strongly dependent on the amount and duration of N additions. At Rosinedalsheden, the annual addition of 20 kg·N·ha^−1^·yr^−1^ for the past 6 years affected neither the abundance, richness, *δ*^15^N, nor the cumulative amount of N retained in EM sporocarps, whereas the addition of 100 kg·N·ha^−1^·yr^−1^ during the same period nearly eliminated EM sporocarps. This short-term response is in contrast to the long-term response observed at Norrliden, where annual additions of N have been going on for the past 40 years. Despite a considerable decrease in the abundance and a shift in the dominant EM sporocarp taxa, long-term addition of N at Norrliden at rates of *ca*. 35 or 70 kg·N·ha^−1^·yr^−1^ did not eliminate C allocation to EM sporocarps. Furthermore, 20 years after the termination of N addition in the highest N treatment (N3T), the abundance, richness, and *δ*^15^N values of EM sporocarps and tree foliage were not different between the control and N3T treatment, indicating a recovery of the functional role EM sporocarps play in the forest N and C cycles after the cessation of long-term N loading.

The contrasting responses of EM sporocarp communities to the two N addition rates at Rosinedalsheden suggest that the response of EM fungi to increasing N availability is more complex than the often assumed constant, negative linear relationship (Arnolds 1991; Lilleskov et al. 2001, 2002a; Kjoller et al. 2012). The abundance and species richness of EM sporocarps was similar between the control and low N treatment, whereas the addition of 100 kg·N·ha^−1^·yr^−1^ nearly eliminated EM sporocarps in the high N treatment. This finding suggests that with increasing amounts of N additions in N-limited boreal forests, there appears to be a threshold that only when reached elicits a negative response in EM fungal communities. Previously, Wallenda and Kottke (1998) suggested a critical load of 35 kg·N·ha^−1^·yr^−1^ as sufficient to cause changes in the abundance of EM sporocarps, which is consistent with our observation of no negative effect of 20 kg·N·ha^−1^·yr^−1^, but a strong negative effect of 100 kg·N·ha^−1^·yr^−1^ at Rosinedalsheden.

Despite a considerable change in EM sporocarp taxa, long-term additions of 35 and 70 kg·N^−1^·ha^−1^·yr^−1^ in both the N1 and N2T treatments; respectively, at Norrliden did not eliminate C allocation to EM sporocarps, although the amount of C allocated to EM sporocarps was small (<100 g·m^−2^). Increasing N additions resulted in higher dominance of *Lactarius* and *Laccaria* species (e.g., nitrophilic species) in the N1 and N2T treatments, which are presumably more successful at using inorganic N sources, have hydrophilic short- and medium-distance or contact exploration types, and thus pose a lower C cost to their host (Hobbie and Agerer 2010; Lilleskov et al. 2011). *Cortinarius* species, on the other hand, were the dominant EM taxa in the control, yet absent from the N1 and N2T treatments. *Cortinarius* species are thought to be more capable of using complex organic N sources (Lilleskov et al. 2002b), possess hydrophobic long-distance exploration types (Hobbie and Agerer 2010), and consequently may require more C from their host (Lilleskov et al. 2011). The notion that changes in EM fungal communities in response to N additions at Norrliden may be the result of taxon-specific differences in N use is consistent with a previous study at the same site that showed significantly higher concentrations of inorganic N in the N1 treatments compared with the control (Högberg et al. 2006). However, these broad generalizations about the source of N used by EM fungi are largely based on laboratory studies and remain untested in the field.

The termination of two long-term N addition treatments at Norrliden provided a unique opportunity to examine the temporal recovery of EM sporocarp production after N saturation. Three years after the termination of N addition in the N2T treatment, the abundance and richness of EM sporocarps was still reduced compared to the control, indicating no immediate recovery of EM sporocarp production. In contrast, EM sporocarp abundance and richness in the N3T treatment was similar to the control, suggesting that within 20 years after the cessation of long-term N additions, C allocation to EM sporocarp production was restored. This finding is in contrast to previous studies that have shown the production of EM sporocarps can be depressed for decades or become extirpated after high N loading (Strengbom et al. 2001; Arnolds 2010). The recovery of EM sporocarps in the N3T treatment was largely the result of the re-occurrence of *Cortinarius* species, which provides further support that *Cortinarius* sporocarps may be a good indicator of N availability in N-limited boreal forest (Brandrud 1995). However, it is worth pointing out that *Lactarius* species were also abundant in the N3T treatment suggesting a long-lasting legacy effect of N additions on EM sporocarp richness.

Results from this study provide evidence for a tight coupling between tree C allocation to and N retention in EM sporocarps and moreover highlight the potential use of *δ*^15^N in EM sporocarps as a relative index of EM fungal sink strength for N. In sites characterized by low N availability, trees relied heavily on EM fungi for N uptake, as indicated by lower foliar *δ*^15^N values and greater differences in *δ*^15^N between EM sporocarps and tree foliage (Δ) (Taylor et al. 2003; Hobbie and Hobbie 2006; Högberg et al. 2011). In exchange for improving N uptake, trees allocated more C to EM sporocarps, which resulted in greater amounts of N retained in EM sporocarps, because of the high demand for N to build fungal biomass, and higher EM community *δ*^15^N values. However, with increasing N availability, trees become less dependent on mycorrhizal fungi for N uptake, as indicated by a reduction in EM root tips (Kjoller et al. 2012), and as a result, N is readily taken up by tree roots. There is little fractionation of N when directly taken up by plant roots (Evans 2001), which in part may explain similar foliar and soil *δ*^15^N values in the ongoing N1 treatment. Additionally, less N transferred by EM fungi would result in reduced N fractionation by EM fungi, which in turn could explain the negative relationship between EM community *δ*^15^N values and increasing N availability. Thus, changes in EM community *δ*^15^N values in response to N additions appear to be the result of less C allocated to and correspondingly less N retained in EM sporocarps and thus may provide a relative index of N retention by EM fungi.

We recognize that differences in EM community *δ*^15^N values among treatments could also reflect changes in dominant EM genera and the depth at which their mycelium occurs. It is well known that *δ*^15^N values increase with soil depth (Nadelhoffer and Fry 1988; Högberg 1997), and more recently, Hobbie et al. (2014) demonstrated that *Cortinarius* species use soil N at deeper depths than *Laccaria* and *Lactarius* species. The observed differences in EM community *δ*^15^N values among N addition treatments may therefore simply reflect changes in dominant EM taxa and where they access N in the soil profile. However, sporocarp *δ*^15^N for individual EM taxa also differed among N addition treatments at Norrliden, and moreover, these differences were positively correlated with changes in their abundance within the different N treatments. Taken together, our results suggest that changes in EM community *δ*^15^N values in response to increasing N availability were not entirely the result of changes in dominant EM sporocarp taxa, but also reflect the demand for N to build fungal biomass (e.g., sporocarps) which is strongly dependent on the amount of C allocated to EM fungi.

Interestingly, 20 years after the termination of N additions in the highest N treatment (N3T), EM community *δ*^15^N and Δ values approached those observed in the control. Furthermore, when expressed as a percentage of the total amount of soil N, the cumulative amount of N retained in EM sporocarps was twice as great in the control and N3 treatment compared with the N1 and N2T treatments. Although the total amount of N retained in EM sporocarp is small, ranging between 0.14 and 0.22 kg·N·ha^−1^·yr^−1^, it is important to point out that EM sporocarps represent only a small fraction of the total EM fungal biomass in natural ecosystems (Sims et al. 2007), and therefore, a much larger portion of N retained in EM fungal biomass would be found in extramatrical mycelium in the soil (Wallander et al. 2004; Nilsson et al. 2012). Indeed, it has been estimated that 2.5–7.5 kg·N·ha^−1^ can be retained in EM mycelium each year, which represents a significant portion of the total amount of soil N retained in European coniferous forests (Nilsson et al. 2012). In addition to representing a large pool of organic N, EM fungal litter is thought to mineralize slowly and so could be important for ecosystem N retention (Langley et al. 2006). Taken together, these findings suggest a recovery of EM functioning after cessation of high N loading and also highlight the potential role EM fungi play in N retention in forest soils (Högberg et al. 2011; Nilsson et al. 2012; Näsholm et al. 2013). However, further studies aimed at determining the decomposition rates of EM fungal litter in the field are needed to better understand the functional role EM fungi play in the forest soil N cycle.

In this study, we choose to focus on EM sporocarps when evaluating the effects of N additions of EM fungi because of their known sensitivity to N availability as well as the relative ease to identify and perform isotopic analyses on individual species. We recognize that EM sporocarps represent only a fraction of total EM biomass (Sims et al. 2007) and that changes aboveground do not necessarily correspond to changes belowground (Lilleskov et al. 2002a,b2002b; Avis et al. 2003; Toljander et al. 2006). Nevertheless, our results are consistent with other studies that have shown a reduction in C allocation to mycorrhizal fungi in response to N additions at high dosage rates or over long periods of time (Brandrud 1995; Wallenda and Kottke 1998; Lilleskov et al. 2002a,b2002b; Avis et al. 2003; Toljander et al. 2006; Parrent and Vilgalys 2007; Lilleskov et al. 2011; Kjoller et al. 2012). Additionally, the strong correlations among cumulative EM sporocarp biomass, EM community *δ*^15^N, and the amount of N retained in EM sporocarps provide evidence that the production and *δ*^15^N of EM sporocarps could be used as relative indicators of the functional role EM fungi play in the forest C and N cycles in light of increasing N availability.

Changes in C allocation to EM fungi in response to N additions could also alter aboveground plant growth through a process known as the mycorrhizal–fungal-induced progressive limitation (PNL) hypothesis (Alberton et al. 2007). According to this hypothesis, greater belowground tree C allocation in N-limited environments is coupled to a higher EM fungal sink strength for N, and consequently, less N is transferred to host plants resulting in a reduction in aboveground plant growth (Nylund and Wallander 1989; Colpaert et al. 1992, 1996; Alberton et al. 2007; Correa et al. 2008). The negative relationships among nitrogen availability, C allocation to and N retention in EM sporocarp biomass, and foliar N concentrations found in this study support this hypothesis and could help explain reduced aboveground tree growth in the control relative to the N1 and N2T treatments as previously described in Högberg et al. (2006). Interestingly, the recovery of C allocation to EM sporocarps in the N3T treatment may provide an explanation for the observed reduction in aboveground tree growth in the N3T treatment after the termination of N additions (Högberg et al. 2006).

## Conclusions

This study clearly demonstrates that the response of EM sporocarps to increasing N availability is complex and is strongly dependent on the amount and duration of N additions. At Rosinedalsheden, the addition of 100 kg·N·ha^−1^·yr^−1^ for 6 years essentially eliminated EM sporocarps, whereas the addition of 20 kg·N·ha^−1^·yr^−1^ did not affect sporocarp production and if anything slightly increased the amount of N retained in EM sporocarps. Although long-term additions of N at Norrliden lead to a change in the dominant EM sporocarp taxa, it did not eliminate EM sporocarp production. Instead, reduced tree C allocation to EM sporocarps in response to long-term N additions was coupled to lower EM community *δ*^15^N values, suggesting a decrease in the functional role of EM fungi as N sinks. However, 20 years after the termination of N additions in the most heavily fertilized treatment, EM sporocarp abundance, richness, community *δ*^15^N, and Δ values were no longer different than the control, suggesting a recovery of the functional role EM fungi play in the forest N cycle. Given the important functional role EM fungi play in forest soil C and N dynamics (Högberg et al. 2011; Nilsson et al. 2012; Clemmensen et al. 2013; Näsholm et al. 2013), a better understanding of the dosage-dependent effects and recovery of EM functioning after N saturation should be a strong future research priority.

## Supporting Information

Table S1 Mean (±SE) δ^15^N of ectomycorrhizal (EM) sporocarps taxa collected from the short-term N addition treatments at Rosinedalsheden during the fall of 2011.

Table S2 Mean (±SE) δ^15^N of ectomycorrhizal (EM) sporocarps taxa collected from the long-term N addition treatments at Norrliden during the fall of 2011.
