# Supplementary material for: Dosage and duration effects of nitrogen additions on ectomycorrhizal sporocarp production and functioning: an example from two N-limited boreal forests
Source: Ecol Evol. 2014 Jul 5;4(15):3015–26. doi: 10.1002/ece3.1145 (PMC4161175; doi:10.1002/ece3.1145)
Supplement: Table S1 — Mean (±SE) δ15N of ectomycorrhizal (EM) sporocarps taxa collected from the short-term N addition treatments at Rosinedalsheden during the fall of 2011. [file ece30004-3015-sd1.docx]

**Supporting Information Table S1** Mean (±SE) δ^15^N of ectomycorrhizal (EM) sporocarps taxa collected from the short-term N addition treatments at Rosinedalsheden during the fall of 2011. Nitrogen addition treatments started in 2006 and consisted of a low and high treatment that received annual additions of NH_4_NO_3_ of 20 and 100 kg N ha^-1^; respectively, and an unfertilized control. Collections were made on a weekly basis between August and September, as this period corresponded with most EM sporocarp emergence. Values in parentheses correspond to the number of isotopic analyses conducted out of the total number of sporocarps collected for each species.

|  | **Nitrogen treatment** | | |
| --- | --- | --- | --- |
| **Ectomycorrhizal species** | **Control** | **Low** | **High** |
| *Boletus pinophilus* Pil. & Dermek | 9.18 (1) |  |  |
| *Chroogomphus rutilus* (Schaeff.) Mill. | 4.88 (1^*^/3) | 0.24 ± 0.36 (3^*^/5) |  |
| *Cortinarius anomalus* (Fr.) Fr. | 6.53 (1^*^/4) |  |  |
| *Cortinarius brunneus* Fr. | 6.70 ± 0.75 (5^*^/118) | 7.07 ± 0.51 (5^*^/56) |  |
| *Cortinarius cinnamomeus* (L.) Fr. | 3.93 ± 0.58 (7^*^/30) | 3.29 ± 0.60 (9^*^/41) |  |
| *Cortinarius delibutus* Fr. | 8.12 ± 4.73 (2^*^/4) | 11.05 ± 3.02 (3^*^/5) |  |
| *Cortinarius evernius* (Fr. : Fr.) Fr. | 6.10 ± 0.30 (8^*^/70) | 5.74 ± 0.67 (6^*^/80) |  |
| *Cortinarius integerrimus* Kühner | 10.46 ± 2.84 (2^*^/6) | 8.03 ± 1.23 (2^*^/14) |  |
| *Cortinarius laniger* Fr. |  | 5.85 (1^*^/2) |  |
| *Cortinarius lucorum* (Fr.) J.E. Lange | 6.23 (1/1) | 5.63 (1/1) |  |
| *Cortinarius mucosus* (Bull.) Cooke | 7.00 (1^*^/2) | 6.76 (1/1) |  |
| *Cortinarius ochrophyllus* (Fr. : Fr.) Fr. | 5.51 (1/1) | 2.77 ± 1.02 (4^*^/13) |  |
| *Cortinarius paleaceus* (Weinm.) Fr. | 5.95 ± 0.23 (3^*^/12) |  |  |
| *Cortinarius semisanguineus* (Fr.) Gill. | 4.14 ± 0.24 (9^*^/48) | 4.35 ± 0.21 (11^*^/139) |  |
| *Cortinarius speciosissimus* Kühner & Rom. | 3.87 ± 0.01 (3^*^/35) | 4.62 (1^*^/5) |  |
| *Cortinarius talus* Fr. | 2.93 (1/1) | 4.31 ± 0.47 (3^*^/11) |  |
| *Cortinarius traganus* Fr. | 8.93 ± 1.03 (6^*^/8) | 9.84 ± 0.88 (4^*^/11) |  |
| *Laccaria bicolor* (Maire) Orton |  |  | 0.46 ± 0.13 (2^*^/12) |
| *Lactarius rufus* (Scop.) Fr. | 3.06 ± 0.24 (8^*^/82) | 2.54 ± 0.15 (12^*^/149) |  |
| *Leccinium versipelle* (Fr.) Snell |  | 11.00 ± 0.60 (2^*^/3) |  |
| *Phellodon niger* (Fr. :Fr.) Karst. | 9.14 ± 2.72 (2^*^/6) | 8.51 (1^*^/4) |  |
| *Phellodon tomentosa* L. : Fr.) | 11.77 (1^*^/35) |  |  |
| *Russula integra* L. ex Fr. |  | 2.97 (1^*^/2) |  |
| *Russula luteotacta* Rea. | 3.18 ± 0.41 (2/2) |  |  |
| *Russula roseipes* Secr. Ex Bres. | 2.29 (1^*^/2) |  |  |
| *Suillus variegatus* (Swartz ex Fr.) O. Kuntze | 5.01 ± 0.31 (5^*^/15) | 7.80 ± 0.38 (5^*^/38) |  |

^*^multiple sporocarps of the same species were collected in a plot on a given sampling data and consequently they were pooled prior to isotopic analyses.
