# Supplementary material for: Dosage and duration effects of nitrogen additions on ectomycorrhizal sporocarp production and functioning: an example from two N-limited boreal forests
Source: Ecol Evol. 2014 Jul 5;4(15):3015–26. doi: 10.1002/ece3.1145 (PMC4161175; doi:10.1002/ece3.1145)
Supplement: Table S2 — Mean (±SE) δ15N of ectomycorrhizal (EM) sporocarps taxa collected from the long-term N addition treatments at Norrliden during the fall of 2011. [file ece30004-3015-sd2.docx]

**Supporting Information Table S2**. Mean (±SE) δ^15^N of ectomycorrhizal (EM) sporocarps taxa collected from the long-term N addition treatments at Norrliden during the fall of 2011. Nitrogen addition treatments started in 1971 and consisted of three annual addition rates of NH_4_NO_3_: 35 (N1), 70 (N2T), and 110 kg N ha^-1^ yr^-1^ (N3T), respectively, and a control (N0). In 1991, after 20 years of N additions, the highest N treatment (N3T) was terminated and in 2009 N addition in the N2T treatment was terminated. Collections were made on a weekly basis between August and September, as this period corresponded with most EM sporocarp emergence. Values in parentheses correspond to the number of isotopic analyses conducted out of the total number of sporocarps collected for each species.

|  | **Nitrogen Treatment** | | | |
| --- | --- | --- | --- | --- |
| **Ectomycorrhizal species** | **N0** | **N1** | **N2T** | **N3T** |
| *Chroogomphus rutilus* (Schaeff.) Mill. | 4.03 ± 1.14 (4/7) |  |  | 6.55 ± 0.62 (3/3) |
| *Cortinarius brunneus* Fr. | 7.22 ± 0.28 (4^*^/20) |  |  | 9.62 ± 0.36 (3^*^/25) |
| *Cortinarius cinnamomeus* (L.) Fr. | 6.85 ± 1.11 (3^*^/9) |  |  | 6.55 ± 0.44 (8^*^/22) |
| *Cortinarius evernius* (Fr.) | 8.38 ± 0.47 (6^*^/38) |  |  | 9.24 ± 0.35 (3^*^/23) |
| *Cortinarius mucosus* (Bull.) Cooke | (2) |  |  |  |
| *Cortinarius semisanguineus* (Fr.) Gill. | 6.33 ± 0.19 (9^*^/70) |  |  | 9.27 ± 0.40 (4^*^/52) |
| *Cortinarius speciosissimus* Kühner & Rom. | 8.63 (1^*^/5) |  |  |  |
| *Laccaria bicolor* (Maire) Orton |  |  | 2.04 ± 1.11 (3^*^/5) |  |
| *Laccaria laccata* (Scop. Ex Fr.) Bk & Bk) |  |  | 0.70 ± 0.30 (5^*^/48) | -1.22 ± 0.37 (2^*^/7) |
| *Lactarius fuliginosus* Fr. | 5.18 (1/1) |  |  |  |
| *Lactarius olivaceoumbrinus* Hesler & A.H. Smith |  |  |  | 2.30 ± 0.14 (3^*^/20) |
| *Lactarius rufus* (Scop.) Fr. | 4.29 ± 0.31 (3^*^/4) | 3.14 ± 0.61 (3^*^/18) | 2.95 ± 0.43 (6^*^/22) | 3.24 ± 0.21 (11^*^/80) |
| *Paxillus involutus* (Batsch) Fr. |  | 6.05 ± 0.55 (3^*^/4) | 6.90 ± 0.76 (2^*^/4) | 3.88 ± 0.19 (4/5) |
| *Phellodon niger* (Fr. :Fr.) Karst. |  |  |  | (1) |
| *Russula aeruginea* Fr. |  | 3.56 ± 1.33 (2/2) | 3.87 ± 0.62 (6^*^/19) | 5.43 ± 0.41 (6^*^/9) |
| *Russula delica* Fr. | 6.70 ± 0.02 (2^*^/5) |  |  |  |
| *Russula integra* L. ex Fr. | 2.55 (1^*^/3) |  |  |  |
| *Russula luteotacta* Rea | 3.88 (1^*^/2) |  |  |  |
| *Russula vinosa* Lindbl. | (9) |  |  |  |
| *Suillus variegatus* (Swartz ex Fr.) O. Kuntze | 7.86 (1/1) |  |  | 6.36 ± 0.19 (2^*^/16) |
| *Xerocomus subtomentosus* (L : Fr) Quél. |  | 5.17 ± 0.37 (6^*^/16) | 5.17 ± 0.61 (2^*^/2) |  |

^*^multiple sporocarps of the same species were collected in a plot on a given sampling data and consequently they were pooled prior to isotopic analyses.
